# Community strengthening and mental health system linking after flooding in two informal human settlements in Peru: a model for small-scale disaster response

**DOI:** 10.1017/gmh.2017.33

**Published:** 2018-03-12

**Authors:** C. Contreras, M. Aguilar, B. Eappen, C. Guzmán, P. Carrasco, A. K. Millones, J. T. Galea

**Affiliations:** 1 Socios En Salud, Lima, Peru; 2 Department of Global Health and Social Medicine, Harvard Medical School, Boston, USA

**Keywords:** Disaster, flood, interventions, mental health, mental health accompaniment

## Abstract

**Background:**

Mental health is an important factor in responding to natural disasters. Observations of unmet mental health needs motivated the subsequent development of a community-based mental health intervention following one such disaster affecting Peru in 2017.

**Methods:**

Two informal human settlements on the outskirts of Lima were selected for a mental health intervention that included: (1) screening for depression and domestic violence, (2) children's activities to strengthen social and emotional skills and diminish stress, (3) participatory theater activities to support conflict resolution and community resilience, and (4) community health worker (CHW) accompaniment to government health services.

**Results:**

A total of 129 people were screened across both conditions, of whom 12/116 (10%) presented with depression and 21/58 (36%) reported domestic violence. 27 unique individuals were identified with at least one problem. Thirteen people (48%) initially accepted CHW accompaniment to government-provided services.

**Conclusions:**

This intervention provides a model for a small-scale response to disasters that can effectively and acceptably identify individuals in need of mental health services and link them to a health system that may otherwise remain inaccessible.

## Background

Beyond the physical effects of the disaster, a minority of people may experience mental health problems, either immediately or over the span of subsequent years (Norris *et al.*, 2002). Inattention to the mental health needs of those affected by the disaster may affect their interpersonal relationships and weaken the social, cultural, and physical resources of their communities. A recent systematic review reinforced the importance of mental health case identification and triage in responding to disasters given that interventions such as psychological first aid, psychological debriefing, and crisis counseling lack rigorous empirical evaluation and may even cause harm (North & Pfefferbaum, 2013). Common disaster response efforts consist of diverse strategies for intervention (Gordon, 2004; Pineda & Lopez, 2010). Some approaches are explicitly focused on the provision of traditional clinical services, whereas others aim to promote healing through *meaning-making* (see Kalayjian & Eugene, 2010), extending the work of Victor Frankl, influenced by his personal experiences and his witnessing of trauma in Nazi concentration camps (Frankl, 1959). It is important for disaster response efforts to support both individual- and community-level recovery (Ursano *et al.*, 2007). Recent efforts in Haiti and Nepal have demonstrated the value of approaches that integrate community-based intervention, case-identification, and health systems strengthening in meeting the mental health needs following natural disasters (Raviola *et al.*, 2012; Sherchan *et al.*, 2017). Such an integrated approach is consistent with the broad aims of the Inter-Agency Standing Committee's Guidelines on Mental Health and Psychological Support in Emergency Settings (IASC, 2007).

Peru, a country characterized by the ‘El Niño,’ ‘*The Child,’* climatic patterns, has been subject to devastating flooding in its history, most notably in the disasters of 1982–1983 and 1997–1998 (SENAMHI, 2015). In 2017, Peru was affected by a weather pattern leading to widespread flooding and mudslides known as ‘El Niño Costero,’ ‘*The Costal Child’* (BBC Mundo, 2017), that caused countless material and health consequences. An estimated 1 138 619 people were affected; 235 806 people were considered victims; and 145 people died as a result (El Comercio, 2017). In Lima, only 24.3% of those with mental illnesses actually access mental health services whereas the remaining 75.7% do not access care due to various cultural and economic barriers (INSM, 2013).

A community health organization based in Lima, Peru [Socios En Salud (SES)] initiated a disaster response effort, attending to the clinical and socioeconomic issues of affected communities through a series of medical brigades. These initial efforts made clear the need for psychological support on an individual and group level, which is the subject of the present report. In Lima, free government-sponsored mental health services exist; however, they remain inaccessible to many eligible citizens due to factors such as stigma, geographic distance, and resource scarcity. The present paper describes an intervention to improve access to these services and support community-level psychological resilience several weeks following flooding in two informal human settlements (IHS) in the Lima-metropolitan area.

## Methods

Two IHS affected by the disaster were identified as targets for the intervention, in the eastern (IHS-East) and northern (IHS-North) outskirts of Lima, affected by the Rímac River and Chillón River, respectively. Prior to the mental health intervention, a multidisciplinary team from SES-provided clinical and socioeconomic support through medical brigades. A team of psychologists in the organization subsequently developed the focused mental health intervention described herein.

Initial meetings with community leaders and experience from the medical brigades allowed the team to identify the most problematic and common problems following the disaster, depression, and domestic violence. The team then trained community health workers (CHWs) and volunteers to support the mental health intervention. The intervention was structured as a ‘mental health fair’ that consisted of three components: (1) screening for depression and domestic violence; (2) implementing activities for children designed to strengthen their social and emotional skills and diminish stress; and (3) involving adults in the community in a ‘theater forum’ facilitated by the team psychologists that invited community participation in acting out life experiences and practicing conflict resolution.

A cut-off score of 15 or higher on the Patient Health Questionnaire 9-item instrument (PHQ-9) was used to identify individuals with depression (Kroenke *et al.*, 2001). Scores of 15–19 and scores > 19 were considered a positive screen for moderate to major depression or severe major depression, respectively. Reports of any form of abuse in the past year on section 7 of the World Health Organization Multi-Country Violence Questionnaire (MCVQ) were used to identify individuals with domestic violence (WHO, 2005). Only women were screened for domestic violence.

Once depression or domestic violence was identified, one of four previously trained CHWs under the supervision of the team of psychologists linked the affected people to appropriate government health services for ongoing care, providing in-person accompaniment to appointments and covering the costs of transportation for a period of two months.

For individuals with depression, the team coordinated care by health professionals in the Ministry of Health clinics and Community Mental Health Centers. The Ministry of Health clinics are staffed by general practitioners and nurses. These clinics can refer to the Community Mental Health Centers which specialize in mental health care and are staffed by clinical psychologists, psychiatrists, and nurses. For individuals with domestic violence, the team coordinated care with an Emergency Women's Center, operated by the Ministry of Women and Vulnerable Populations, which have multidisciplinary teams that support victims of domestic violence with specialized psychological care and support navigating appropriate legal services. As such, those with both domestic violence and depression were not additionally referred to the Ministry of Health clinics. However, the team respected any preference to receive care at the Ministry of Health clinic instead of the Emergency Women's Center. Individuals who did not have public insurance were accompanied to register for public insurance, and individuals who had other insurance were accompanied to their respective clinics, which are not directly operated by the Ministry of Health.

## Results

In total, 129 people were screened, 116 with the PHQ-9 and 58 with the MCVQ. Of those screened, 12 (10%) presented with depression and 21 (36%) reported domestic violence. 27 unique individuals (21%) screened positive for depression or domestic violence, of whom 13 (48%) accepted CHW accompaniment to some government service.

The rates of depression and domestic violence are presented in Table 1. Several individuals experienced depression and domestic violence, as presented in Table 2, and may have sought care for only one of these problems. Individuals who screened positive for domestic violence and accepted accompaniment did not necessarily attend the Emergency Women's Center services but may have only accepted accompaniment to the Ministry of Health clinic.

**Table 1.** Rates of depression and domestic violence encountered

	Informal human settlements (IHS)-East		IHS-North		Total	
	*N*	%	*N*	%	*N*	%
Total people screened	95	100	34	100	129	100
Men	19	20	6	18	25	19
Women	76	80	28	82	104	81
Depression						
People screened for depression	83	100	33	100	116	100
Depression cases identified	6	7	6	18	12	10
Moderate depression (PHQ-9: 15–19)	6	7	5	15	11	9
Severe depression (PHQ-9: 20+)	0	0	1	3	1	1
Domestic violence						
People screened for domestic violence	32	100	26	100	58	100
Domestic violence cases identified (any form)	9	28	12	46	21	36
Psychological abuse (past year)	8	25	8	31	11	19
Physical abuse (past year)	6	19	8	31	8	14
Sexual abuse (past year)	2	6	3	12	5	9

**Table 2.** Acceptance of community health worker (CHW) accompaniment by problem and community

Problem detected		People with problem	People accepting help	% accepting help
Informal human settlements (IHS)-East	Depression only	4	2	
	Depression and domestic violence	2	2	
	Domestic violence only	7	2	
Total IHS-East		13	6	46
IHS-North	Depression only	2	2	
	Depression and domestic violence	4	3	
	Domestic violence only	8	2	
Total IHS-North		14	7	50
Both communities	Depression only	6	4	67
	Depression and domestic violence	6	5	83
	Domestic violence only	15	4	27
Total both communities		27	13	48

In IHS-East, 83 residents were screened for depression (19 men and 64 women), of whom six (7%) presented with moderate depression. Four of these six (66%) accepted CHW accompaniment to government services. Thirty-two women were screened for domestic violence, of whom nine (28%) reported some form of maltreatment (eight psychological, six physical, and two sexual). Four of these women (44%) accepted CHW accompaniment to a government service.

In IHS-North, 33 people were screened for depression (six men and 27 women), of whom six (18%) presented with moderate or severe depression. Five of these six (83%) accepted CHW accompaniment. Twenty-six women were screened for domestic violence, of whom 12 (46%) reported some form of maltreatment (eight psychological, eight physical, and three sexual). Five (42%) accepted CHW accompaniment to a government service.

## Discussion

Through the course of the post-disaster response efforts, our organization encountered a great number of people in emotional distress related to the disaster, gender inequality, and social class. For this reason, our team developed a strategy to intervene in the affected zones for whom the existing health system was not sufficient due to gaps such as limited economic means to seek care, poor knowledge of the available government services, and negative attitudes about care-seeking of family members.

To overcome these gaps, SES decided to support two IHS with scarce resources that were believed to have serious mental health problems. These mental health problems were not necessarily the result of the disaster but rather were more likely symptoms exacerbated in this period or previously undetected. Even so, they merited timely attention. The core of this intervention was an accompaniment to government services by CHWs, demonstrating the value of trained peer-supports who can attend to various problems and partake in solutions appropriate to the community.

The presented findings demonstrate that these communities have insufficient access to mental health services to attend to urgent needs, which may exist with or without a disaster. The post-disaster response has shed light on the inadequacy of the health system to meet the needs of these disadvantaged populations.

This intervention successfully linked inhabitants of these IHS to government mental health resources by identifying problems, coordinating care, providing in-person accompaniment, and covering the prohibitive costs of transportation. However, in this article, we do not present data on the individuals’ actual utilization and continuity of care, which may be lower than the rate of those accepting accompaniment. The results may particularly inflate the true utilization of the Emergency Women's Center as the count of participants accepting services include several who only sought attention at the Ministry of Health clinic. Even so, the high burden of mental health problems encountered in these communities suggests that community-supported interventions after disasters can provide opportunities to facilitate access to care. That only 48% of those identified with depression or domestic violence even accepted accompaniment to government services reflects the existence of additional barriers to accessing care, especially for accessing domestic violence services. Yet, that same figure suggests that for many individuals, this intervention was considered an acceptable and effective form of support following the disaster.

## Conclusion

After Peru's recent natural disaster, the SES team rapidly planned and executed a response to the unattended mental health problems in two vulnerable IHS in the outskirts of Lima. Given the barriers in recognizing mental health problems in these communities, we realized that case identification and CHW accompaniment to free government services would be an important improvement in the access to mental health care, in conjunction with community-based activities. Thirteen people in need of attention related to depression and/or domestic violence were able to access care as a result of our small-scale intervention.

The communities’ openness to external support following a disaster presented the opportunity to carry out an acceptable mental health intervention coordinated with local community leaders. This intervention is an example of an effective model to identify mental health problems in a community setting and accompany those in need of government services despite the barriers faced by these vulnerable populations.

Given the large cultural and economic gaps to access mental health services, it is necessary to implement alternative, community-based models that assure that populations with poor access to the traditional health system can receive care. We present a model that could be extended to other community settings to overcome barriers to accessing mental health care.
